# Uptake of Bayesian robust information borrowing in Phase II clinical trials: a scoping review

**DOI:** 10.1186/s12874-026-03052-3

**Published:** 2026-11-05

**Authors:** Vivienn Weru, Silvia Calderazzo, Leonhard Held, Manuel Wiesenfarth, Annette Kopp-Schneider

**Affiliations:** 1 Division of Biostatistics, German Cancer Research Center (DKFZ), Heidelberg, Germany; 2 Medical Faculty, Universität Heidelberg, Heidelberg, Germany; 3 Epidemiology, Biostatistics & Prevention Institute (EBPI), University of Zurich, Zurich, Switzerland; 4 Cogitars GmbH, Heidelberg, Germany

**Keywords:** External information, Robust mixture prior, Power prior, Bayesian hierarchical model, Effective sample size, Basket trials

## Abstract

**Background and objective:**

Incorporation of external information in clinical study designs has received increasing attention in recent years. When external data can be considered consistent with the data collected in the current trial, efficiency gains and a reduction in the required sample size can be achieved. This makes it particularly attractive when, *e.g*., recruitment of patients is difficult. However, unexpected inconsistency cannot be ruled out, and various robust borrowing approaches have been developed to limit potential losses. These include, for example, the power prior, meta-analytic predictive prior, robust mixture prior, and Bayesian hierarchical model. However, their use in actual clinical trials remains underexplored. In this scoping review, we investigated the use of external information in Phase II clinical trials.

**Methods:**

Publications were identified by literature search in PubMed central, the Cochrane Central Register of Controlled Trials and complemented with a citation search in some of the main methodological papers on Bayesian borrowing in clinical trials.

**Results:**

Of the 1282 articles retrieved, *N* = 37 were eventually included. We investigated, among others, whether and how the suitability of external data was assessed, the borrowing method used, and the specification and justification of tuning parameters that influence the degree of borrowing. Fixed downweighting was found to be the most commonly used method for external information borrowing, with only slightly more than a third of the studies providing details on its implementation.

**Conclusion:**

Overall, we found poor reporting of various aspects relating to Bayesian borrowing, signaling a gap between the methodological developments and the practical uptake and reporting of such methods.

**Supplementary information:**

The online version contains supplementary material available at https://doi.org/10.1186/s12874-026-03052-3.

## Introduction​​

The use of external data to augment clinical trial information has gained popularity in recent years. Such an approach is typically referred to as ’external information borrowing’ and holds the potential to significantly increase the efficiency of a given trial. Areas in which borrowing has seen increasing application are those in which there is interest or need to reduce the sample size, *e.g*., pediatric trials, control or placebo arms, or in the analysis of subgroups, for example, multiple study arms in basket trials. Basket trials are a specific type of clinical trial where a single therapy is evaluated across different disease types with a common biomarker (see, *e.g*., [1–3]). In such a setting, rather than conducting multiple independent trials, only one study protocol is needed.

External data to be incorporated into the current trial requires careful selection, and guidelines have been provided to aid in this selection, for example, by Pocock [4]. In addition, when covariates are available, regression or propensity score approaches can be used to adjust for measured confounding [5–10]. However, unobserved heterogeneity between current and external data can still be present, and for this, robust Bayesian and frequentist methods have been developed (*e.g*., [5, 11–15]) that downweight external information in case of such heterogeneity. Although fixed downweighting approaches are not considered “robust" as they do not adapt to unforeseen prior-data conflict, we included them in this review since they do acknowledge potential heterogeneity and adjust beforehand for this.

Although many methods have been developed to facilitate external data borrowing, their use in actual clinical trials is understudied. Systematic reviews are available that have focused on different aspects of Bayesian methods in clinical trials. For example, the systematic review of Barret et al. [16] investigated the type of priors used in Phase II and III randomized clinical trials, as well as the use of decision thresholds and whether such thresholds were pre-specified. In their systematic review, Ferreira et al. [17] investigated reporting of replicability items in Phase III clinical trials, focusing on BayesWatch [18] and ROBUST [19] guidelines. The recent narrative review of Turner et al. [20] investigates Bayesian analysis in confirmatory trials, exploring aspects such as the reason for choosing Bayesian methods. The review identifies information borrowing as one for the reasons for choosing Bayesian designs. We focus on Phase II studies in this review as the more exploratory nature of Phase II lends itself naturally to the use of external information, and this has not been investigated systematically so far, to the best of our knowledge. We searched for Phase II clinical trials that use robust Bayesian borrowing. We investigated their reporting of the borrowing method used and respective parameter settings. We focused on a scoping review to highlight gaps in current knowledge concerning all potential aspects related to the use of Bayesian robust borrowing approaches.

## Methods

The objective of this scoping review is to investigate the use and reporting of Bayesian robust borrowing methods in clinical trials, with a focus on Phase II trials. The review followed the PRISMA guideline and the PRISMA-ScR [21] checklist is provided as supplementary material.

### Data sources

A search of the literature was performed in PubMed Central and the Cochrane Central Register of Controlled Trials until 22 May, 2025. The search terms are listed in Supplementary Appendix A. In addition, a search for citations in relevant statistical methodological articles was done to identify clinical trials using these methods. The list of the methodological articles whose citations were searched is included in Table A.3.

### Inclusion and exclusion criteria

We included Phase II clinical trials with a publication in English that involved robust Bayesian information borrowing for the analysis of the primary endpoint. Trials that used Bayesian information borrowing without discounting or consistency checks based on any of Pocock’s criteria and/or available covariates were excluded. Also excluded were studies that used only non-informative/weakly informative priors or used robust Bayesian information borrowing only for sensitivity analyses or re-analyses of a past trial.

All titles and abstracts were independently screened by VW and SC, and the relevant full-texts were thereafter independently reviewed. Conflicts were discussed by jointly reviewing relevant information and texts, including all available supporting documents such as the trial protocol and statistical analysis plan in the case of the full-text screening.

### Data extraction

Data were extracted from the articles by VW using a standardized extraction form. SC checked the data for a random 21.6% (*n* = 8) of the articles (based on alphabetical author order) and an additional 8.1% (*n* = 3) in which the information required for extraction was ambiguous. The complete list of data extracted is given in Table A.4. The extracted data were descriptively analyzed using R (version 4.3.0).

## Overview of key aspects on borrowing external data

When borrowing external data, various key aspects must be considered. These aspects are outlined below. A detailed methodological review for each of these aspects is provided in Supplementary Appendix B.

### External data

In borrowing data, various aspects of the external data need to be considered: the source of the borrowed data, suitability of the external data, and target of borrowing. The sources of borrowing can be external or internal. External sources include previous trials, Real-World evidence (RWE) or information elicited from experts. Internal sources include borrowing between arms within a trial, for example, in basket trials. Distinguishing between different sources of external data is important as different assumptions, for example when evaluating operating characteristics, may be applicable depending on the source of data. With respect to the target of borrowing, external information may be borrowed for the control arm of a study, leading to so-called hybrid-control designs, or the external information may be available on the treatment effect directly. Alternatively, information can be borrowed between the treatment arms of the same trial. Another key point is that the external data need careful selection. Pocock [4] outlined six conditions for comparability, including that treatment and its evaluation, as well as patient eligibility, must be the same between external and current studies. The detailed list of Pocock’s conditions is provided in Supplementary Appendix B.3.

### Borrowing methods and assessment

Even if the external data has been carefully selected, heterogeneity between the current and external information may still be present. Different methods have, therefore, been proposed for incorporating the external data that either adaptively or non-adaptively downweight such data to mitigate its impact if such heterogeneity is present. These include the power prior approach [5, 22], the meta-analytic predictive (MAP) prior [11], the robust mixture prior [12], and Bayesian hierarchical models [23, 24] among others. A detailed description of these borrowing methods is provided in Supplementary Appendix B.4.

All borrowing methods rely on parameter settings that regulate the amount of information borrowed. The power prior downweights the likelihood of the external data according to a power parameter, while for Bayesian hierarchical models, the between-study variance parameter determines the degree of borrowing such that if it is zero, all the data is pooled, while if it goes to infinity, no data is borrowed. The MAP prior is again based on a hierarchical model, but external data is summarized into a predictive distribution which is then used as the prior for the current study. The robust mixture prior is a weighted combination of an informative prior, derived from the external data, and a more dispersed component to robustify the prior in case of inconsistencies between the current and external data. Specification of the prior weight as well as parameters for the robust component is, therefore, also needed.

Adjusting for covariates, *e.g*., through linear regression or matching approaches, to correct for measured confounding between the current and external data can also be considered. Nonetheless, the dynamic borrowing methods above help remedy problems associated with unmeasured confounding.

#### Effective sample size (ESS)

The informativeness of a prior can be translated into a more intuitive metric called the prior effective sample size (ESS) which represents, in simple cases, how many observations the prior is worth. In simple settings, the ESS can be easily obtained. For example, for a binomial likelihood with a conjugate *Beta*(*a*, *b*) prior, the ESS is easily obtained as (*a* + *b*). For more complex settings, different approaches have been suggested for calculating the ESS of a prior, with computations becoming non-trivial for conjugate mixture priors and non-conjugate priors. A description of the common ESS methods is provided in Supplementary Appendix B.5. For some prior distributions, different methods might lead to different ESS values. It is, therefore, essential to report the method used to obtain the ESS, especially for non-conjugate settings or with conjugate mixture priors. This has been shown in more detail by Neuenschwander et al. [25], where the authors compare the ESS obtained using different methods, among them the approach by Morita et al. [26] and the expected local-information-ratio (ELIR) approach [25]. In one example with normal data and a Student-t prior, a non-conjugate setting, the authors show the ESS differing considerably for small degrees of freedom for the Student-t prior, and decreasing (increasing) when using the Morita approach (ELIR approach) as the degrees of freedom increase.

With dynamic prior distributions that adapt to prior-data conflict, the ESS of the prior would be different once the data is observed. Wiesenfarth and Calderazzo [27] propose the conflict-sensitive effective current sample size (ECSS) approach that takes into account prior-data conflict to quantify the impact of the prior. The difference in the prior and posterior ESS can also provide an indication of data-adaptation and prior discounting due to conflict. Reporting such quantities is important since, for example, if a robust mixture prior with equal prior weights for the informative and robust components was used and the weights were then updated once the data are observed to, say, 0.8 and 0.2 respectively, the latter contains a different amount of information from the initial specification.

### Reporting guidelines in Bayesian analysis

Clear reporting of Bayesian analysis, as with any statistical analysis, is crucial to ensure understanding and transparency of the analysis to readers. When an informative prior is used, a clear explanation of the choice of prior is needed. To aid in standardized reporting of Bayesian analyses, several guidelines have been developed. These include ROBUST (Reporting Of Bayes Used in clinical STudies) [19], BayesWatch [18] and BARG (Bayesian Analysis Reporting guidelines) [28]. More details on these guidelines are highlighted in Supplementary Appendix B.6. None of the above guidelines, however, is targeted to our application of information borrowing in clinical trials. When using Bayesian borrowing methods, for example those outlined in the Borrowing methods and assessment section, reporting and justification of corresponding tuning parameters and assumptions is essential.

## Results

### Characteristics of the trials

**Fig. 1:**
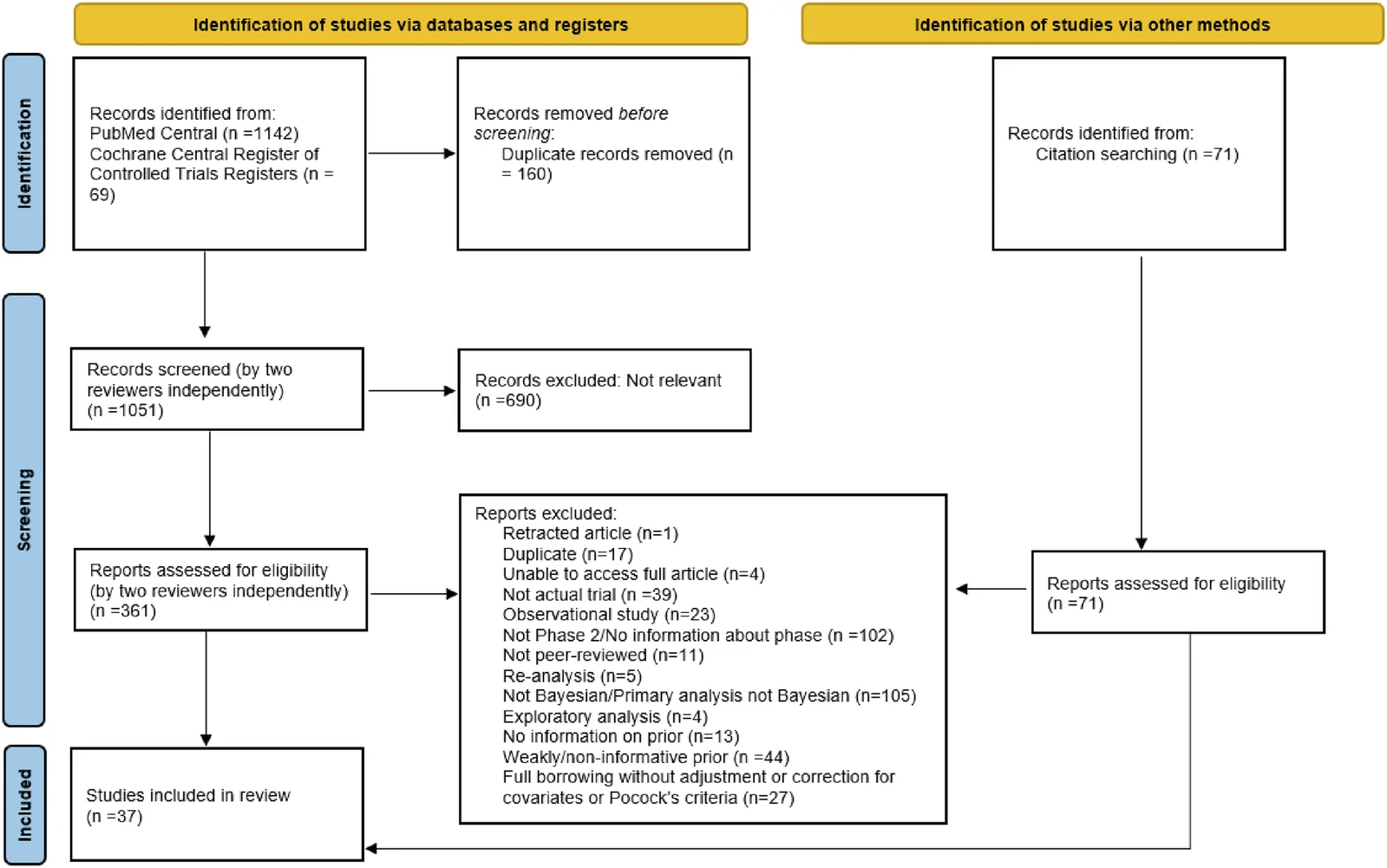
PRISMA flow-diagram of the articles selected and included

Of the 1282 articles retrieved, *N* = 37 were finally included. Figure 1 shows the PRISMA flow chart of the articles selected and included. The excluded reports are listed by the order of exclusion. The list of included articles is provided in Supplementary Appendix C.

To obtain the relevant information for this review, we also checked the supplementary material of the articles, as well as clinicaltrials.gov for the accompanying protocols. Of the included articles, 4 (10.8%) were published before 2015, 18 (48.6%) between 2015 and 2020, and 15 (40.5%) after 2020. Moreover, 86.5% (*n* = 32) were reports while 13.5% (*n* = 5) were protocols of yet unpublished studies. Most of the trials were conducted in oncology (40.5%, *n* = 15), 16.2% (*n* = 6) were in autoimmune diseases, while 10.8% (*n* = 4) were in infectious diseases, with 2 of those investigating COVID-19. Of the included trials, 37.8% (*n* = 14) had a master protocol, mainly basket trials (*n* = 9).

### Key aspects on borrowing external data

As mentioned above, various key aspects must be considered when borrowing external data. In the following, we summarize their distribution in the reviewed studies.

#### Source of borrowed data

In this review, we distinguish between studies that borrow information from external and internal data sources. A majority of the trials borrowed data external to the current trial (62.2%, *n* = 23). Of these, 78.3% (*n* = 18) explicitly cited the sources of the external data while 21.7% (*n* = 5) did not. Seven (30.4%) of the articles that borrowed data external to the current trial explicitly mentioned the source in the protocol, 13.0% (*n* = 3) did not, while it was unclear for 56.5% (*n* = 13) of the articles, mainly due to the lack of availability of the protocol online. External data can come from different sources, *e.g*., historical/concurrent trials, real-world evidence (RWE), or expert opinion. Of the articles citing the source of the external data, none used RWE or elicited the prior information from experts. The studies that borrowed internal information were mainly basket trials.

#### Target of borrowing

Most of the trials borrowed data to the control arm (56.8%, *n* = 21), while 32.4% (*n* = 12) borrowed between arms of the same trial, with the latter mainly being basket trials. When borrowing information on the treatment effect, one study borrowed separately information on the treatment and control arm parameters [29], one borrowed information on the control arm and the treatment effect [30], while two trials only borrowed information on the treatment effect [31, 32], one of those [32] being a single-arm trial.

#### Assessment of external data suitability

As discussed in the introduction, external data need careful selection. Table 1 shows a summary of the approaches used to assess the suitability of the external data. In relation to Pocock’s criteria, the majority of the trials (73.9% (*n* = 17)) assessed at least one of the conditions. One study [33] stated that prior-data conflict would be assessed using graphical displays, one performed pair-matching [34], while one study [35] performed a prior-predictive check to assess the concordance between the data and the prior. Details were not provided for 21.7% (*n* = 5) of the studies.

**Table 1:** Assessment of data suitability for trials that borrowed external information (*N* = 23)

Assessment of the suitability of external data	*n* (%)
Pocock’s criteria	
Same treatment	17 (73.9)
Same patient eligibility	4 (17.4)
Same treatment evaluation	15 (65.2)
Same patient characteristics	7 (30.4)
Same organization and clinical investigators	0 (0)
No other indications	1 (4.3)
Used graphical displays	1 (4.3)
Used pair-matching	1 (4.3)
Used prior-predictive check	1 (4.3)
Not mentioned	5 (21.7)

Note some categories are not mutually exclusive. The detailed list of Pocock’s conditions is provided in Supplementary Appendix B.3

#### Borrowing method used

As mentioned in the Borrowing methods and assessment section​, different methods can be used to incorporate external data. Below we present the borrowing methods identified in the reviewed studies. Again, we distinguish between studies that borrowed data external to the current trial and that borrowed internally. Table 2 shows a summary of the borrowing methods used. The majority of the trials (39.1%, *n* = 9) that borrowed externally used a fixed downweighting approach, with two studies [29, 30] explicitly mentioning the fixed power prior​. The robust mixture prior was used in 21.7% (*n* = 5) of the studies [30, 36–40], for three of which the informative component of the mixture prior was a meta-analytic predictive (MAP) prior. One study [30] used an approach that combined the mixture prior and a power prior to incorporate an aggregate treatment effect as well as individual historical control data, respectively. Three trials used the MAP prior alone [41–43] without robustification.

**Table 2:** Borrowing method used (*N* = 37)

Methods used by trials that borrowed externally to current study	*n* (%)
Fixed downweighting/fixed Power prior	9 (39.1)
Robust mixture prior (w/ or w/o Meta-analytic predictive (MAP))	5(21.7)
Meta-analytic predictive (MAP) (without robustification)	3(13.0)
Bayesian hierarchical model	1(4.3)
Robust MAP and fixed Power prior	1(4.3)
Matching external control to current treatment based on covariates	1(4.3)
Full borrowing with covariates adjustment	1 (4.3)
Dynamic borrowing/Robust assessment but unclear which method	2 (8.7)
Methods used by trials that borrowed from within the same trial	*n* (%)
Bayesian hierarchical model	10 (71.4)
Fixed downweighting/fixed Power prior	2(14.3)
Fujikawa’s design	1 (7.1)
Dynamic borrowing but unclear which method	1(7.1)

Two trials used covariate adjustment to correct for bias [34, 44]. The majority of the trials that borrowed within the same trial used Bayesian hierarchical models, with a majority of these being basket trials. Of these, four trials [45–48] clustered groups with similar response rates. One basket trial [49] reported using Fujikawa’s design, although it did not provide comprehensive details.

#### Parameters influencing degree of borrowing

All borrowing methods are based on parameters that regulate the degree of borrowing, as summarized in Table B.5. For trials using Bayesian hierarchical models, the majority (63.6%, *n* = 7) did not provide further information on how the heterogeneity parameter was estimated, while the rest assigned an inverse gamma prior to it. Of the trials using fixed downweighting, only 33.3% (*n* = 4) explained how the discounting weight was arrived at. Amongst these, two based the decision on simulations evaluating operating characteristics (specifically Type I error (TIE) rate) and two elicited the weight from experts [31, 35]. For example, in the ODYSSEY trial investigating dolutegravir-based antiretroviral therapy in children with HIV infection [31], the borrowing weight *α* was elicited from 13 experienced pediatricians upfront. Brard et al. [30] used *α* = 0.3 for the power parameter of the fixed power prior, McIlroy et al. [50] predefined a weight of 0.75 for the power prior, while the study of Seccia et al. [29] investigated different power parameters for the power prior (*α* = 1, 0.5, 0).

Only one of the included trials [37] that used the MAP, either alone or robustified, provided comprehensive details on the between-trial heterogeneity parameter prior and the derivation of the MAP prior, including the approximation of the MAP prior by a mixture of conjugate distributions. This study used a half-normal prior for the between-trial heterogeneity parameter. The robust mixture prior requires specification of the prior weight, location and dispersion for the robust component, and these parameters need careful thought [51]. All the studies that used the robust mixture prior did specify the prior weight assigned, with three studies using a prior weight of 0.5 while the other three studies assigned a prior weight of 0.1, 0.95 and 0.90 to the informative component of the mixture. However, 50% (*n* = 3) did not give information on how the prior weight was chosen; two based the decision on simulations that evaluated operating characteristics, while one mentioned that they chose the 50% prior weight to avoid prior domination after considering the size of the historical studies and the placebo group. Regarding the informativeness of the robust component of the mixture prior, only three provided the exact prior specification. For trials with a normal endpoint and using the robust mixture prior, one used the same location as historical data, one had the location at the null hypothesis, while one gave no information.

#### Effective sample size

**Fig. 2:**
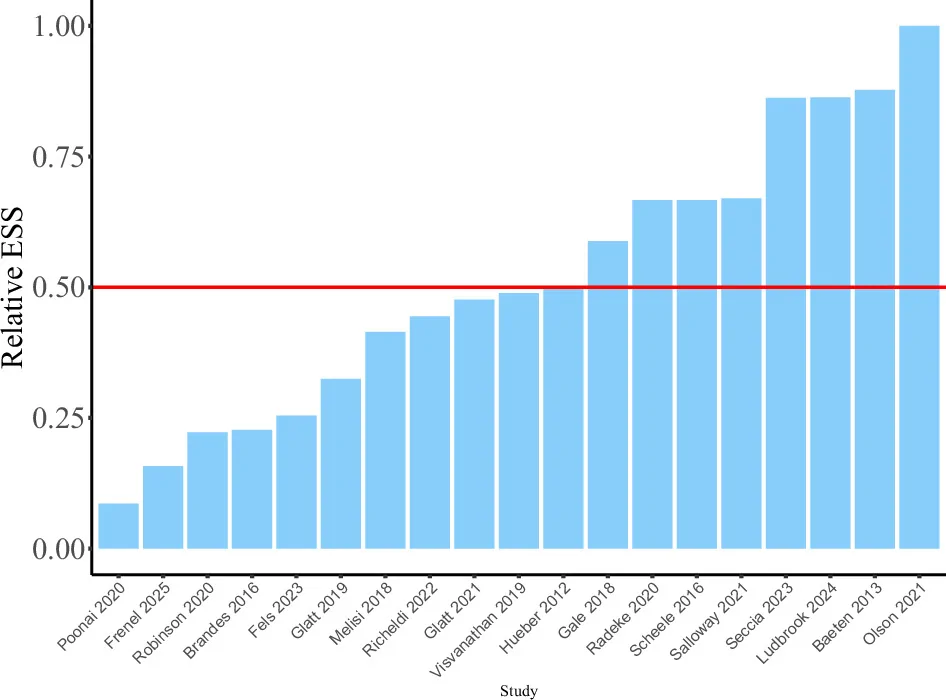
Relative ESS of studies borrowing external data, obtained by dividing the prior ESS by the sum of the prior ESS and current sample size

As outlined in the ESS section earlier​, the ESS provides a more accessible measure of the informativeness of the prior. For trials that borrowed externally to the current trial, 82.6% explicitly stated the ESS or, if not explicitly stated, the ESS can be easily obtained because a conjugate non-mixture model was used. Seven trials (36.8%) explicitly stated the ESS, but since the model was not conjugate, it was not clear which ESS method they used. Figure 2 shows the relative ESS [52]. Of the trials where an ESS could be obtained, eight trials had a relative ESS greater than 0.5. One trial [34] had only historical controls but matched patients to the treatment arm to correct for bias.

#### Success criteria used

In Bayesian designs, one common success criterion is based on posterior probability of the alternative hypothesis. For example, if the alternative hypothesis is *θ* > 0, where *θ* is the treatment effect parameter, a trial would be declared successful if Pr(*θ* > 0∣data) > *κ*, where *κ* is a minimum probability threshold (*e. g*., 95%). When using the posterior probability as success criterion, pre-specification of *κ* is needed. Another approach includes checking whether the posterior credible interval at some pre-specified *κ* includes *θ*_0_, where *θ*_0_ is the null hypothesis value. Most of the studies (86.5%, *n* = 32) used the posterior probability as a success criterion, with one of those relying on a normal approximation for the posterior. Two trials used credible intervals, while the success criterion was unclear for three trials.

#### Essential items to report in Bayesian analyses

Although various guidelines have been proposed for reporting a Bayesian analysis of a clinical study, in this review we focus on a list of seven items known as ROBUST, as outlined in the Reporting guidelines section, pertaining to the prior distribution, analysis, and results. The results are shown in Table 3. For studies that used the Bayesian hierarchical model, details of the prior pertain to the heterogeneity parameter. Specification of the analytical technique and sensitivity analysis on priors were the least reported items (21.6% and 29.7%, respectively). A rationale for the prior specification was provided in 72.9% of the articles, but an explicit specification of the parameter values of the prior was reported in 56.7% of the articles.

**Table 3:** Reporting of ROBUST items (*N* = 37)

Items	*n*	%
Prior distribution		
Specified	21	56.7
Justified	27	72.9
Sensitivity analysis	11	29.7
Analysis		
Statistical model	35	94.6
Analytical technique	8	21.6
Results		
Central tendency	29	78.4
Standard deviation or credible interval	27	72.9

## Discussion

We conducted a scoping review to investigate the uptake of Bayesian robust borrowing in Phase II clinical trials. We evaluated various aspects of borrowing, for example, the borrowing method used and the reporting of associated parameters. A potential limitation of this scoping review is that the search was performed only in PubMed Central and the Cochrane Central Register of Controlled Trials. Nevertheless, this search was complemented by a citation search in the most well-known statistical methodological papers on Bayesian borrowing. Another limitation is that while the screening and full-text assessment was done by two reviewers independently, the data extraction for the final included articles was done by a single reviewer using a standardized extraction form while a second reviewer checked the extracted data for a random sample of the articles and an additional number in which the information required for extraction was ambiguous.

When external data are incorporated into a clinical trial, they need to be carefully selected. Guidelines have been proposed to aid in such selection, for example, Pocock’s criteria [4]. Although some studies indicated that the historical data were obtained from studies similar to the current study, many did not provide additional details.

Different methods can be used to formulate the external data as a prior for the current data. Although fixed borrowing methods are simpler, they preclude adaptation to unforeseen prior-data conflict. However, even though dynamic borrowing methods adapt to prior-data conflict, they still incur losses in terms of estimation and testing performance if moderate conflict is present. Furthermore, most of the dynamic borrowing methods rely on tuning parameters that impact how much information is discarded based on a given observed drift. We found poor reporting of the parameters that regulate the amount of information borrowed in terms of both providing explicit values and rationales for such values. For example, when using the Bayesian hierarchical model, many studies did not give details about the heterogeneity parameter that drives the borrowing. As detailed in Supplementary Appendix B.4, this parameter is crucial. For studies using a fixed downweighting approach, crucial details on the downweighting factor were mainly unavailable. For the robust mixture prior, the prior weight and the robust component drive the borrowing, and these choices need careful thought as detailed in Weru et al. [51]. Again, full details on such parameters were not reported in the majority of the studies. The borrowing parameters can have a dramatic impact on the results. The impact, for example, of a robust mixture prior with 0.1 prior weight on the informative component is markedly different from one with 0.9 prior weight on the same component. Failure to report the borrowing parameters makes it hard for readers to know how much of the external data was included in the posterior inference, especially if the prior ESS was also not reported or could not easily be obtained. Moreover, for dynamic borrowing methods, the initial prior specified adapts to observed prior-data conflict, thereby changing the amount of information the prior is worth. Reporting the prior parametrization is, additionally, important since, for example, with normal data, a normal prior would behave differently from, say, a Student-t prior, in the presence of prior-data conflict. As no standard choices for the parameters are available, explicit reporting is critical.

In addition to the values of the tuning parameters, justification for such choices needs to be provided. When justification was given, the majority of the studies evaluated operating characteristics, followed by an elicitation from clinical experts. In this context, TIE rate and power are the operating characteristics most frequently evaluated. In basket trials, in addition to the basket-specific TIE rate, the family-wise TIE rate is also usually investigated. If a simulation study is used to select a borrowing method or parameter value, appropriate pipelines and reporting should be followed to ensure transparency and fairness for all evaluations [53]. Although operating characteristics are usually evaluated in a frequentist framework, Bayesian metrics have also been proposed [54, 55]. Although none of the studies looked at the latter, it is expected that these evaluations will become more common following the recent FDA draft guidance “Use of Bayesian Methodology in Clinical Trials of Drug and Biological Products Guidance for Industry" [56], which paves the way towards acceptance of the Bayesian paradigm in drug development. Such evaluations will require specification and reporting of additional quantities, most notably the design prior used to generate the simulated data.

The search of the literature was updated on July 17, 2026. With the use of Bayesian borrowing on the rise, nine additional relevant articles were found. As the added evidence did not alter our conclusions, the analyses were not rerun. Of the new articles found, two borrowed internally while the rest borrowed outside the current trial. For the articles where borrowing was external, the source of the borrowed data was not outlined in one of the articles, and in three of the studies, there were no mentions of assessment of the external data suitability. Two used the robust mixture prior approach, one used a MAP prior, two used a fixed downweighting approach, while two used a Bayesian hierarchical model to dynamically borrow across the active treatment arms. One compared the current treatment with a historical placebo group, with the historical placebo group chosen based on various similarity aspects. The borrowing approach was unclear for one. Further, borrowing parameters were unreported in a substantial number of the articles and, when reported, justification on how these were chosen was predominantly unavailable. The list of the additional articles is provided in Supplementary Appendix D.

Although some of the aspects we investigated are specific to clinical trial designs that involve robust borrowing, general guidelines for the reporting of Bayesian analyses also exist. We focused on ROBUST [19], although other guidelines are available. Despite some of the guidelines being available for many years, studies are yet to fully embrace them. Various important considerations and aspects in the context of external data borrowing have also been discussed in ICH and FDA guidelines. For example, in pediatric extrapolation, the ICH guidance E11A [57] outlines important aspects to consider, including evaluating the definition and course of disease between the reference and the target populations, as well as considerations for extrapolation of safety. Further aspects include emphasis on deciding on the sources of information in consultation with regulatory authorities when possible, pre-specification of the borrowing method, and incorporation of sensitivity analyses to evaluate impact of different prior assumptions. The recent FDA draft guidance [56] details the necessary information that should be submitted to the FDA at the design stage of a trial that aims to use Bayesian methods. These include explicitly specifying the prior parametrization. When using informative priors, the FDA draft guidance asks for justification for borrowing and why the available information is considered relevant for the current study. The sources of this information should be clearly reported as well as the techniques used to synthesize the data if the data come from multiple sources. Failure to report the source of the borrowed data makes it impossible to determine the relevance of the included data. The FDA draft guidance highlights some key factors that may influence the relevance of such data, which include “recency” of the data, among others. The borrowing method should be described and justified, as well as any parameters that regulate the amount of information borrowed. The full list can be found in the FDA draft guidance.

The sensitivity of the results to different prior assumptions and degrees of borrowing should also be reported [56, 57] to illustrate how inferences would change if different borrowing assumptions were made. For example, in the post-hoc analysis of the MENSA trial investigating mepolizumab for severe asthma, Best et al. [58] illustrate the impact of the prior weight of the mixture prior with a tipping point analysis, showing the tipping point value of the prior weight after which a conclusion of a statistically significant treatment benefit of mepolizumab would be achieved. Since Bayesian methods can be expected to be increasingly used in the future in clinical trials, comprehensive reporting is required. For general Bayesian reporting, guidelines are already available, e.g., BayesWatch [18], BARG [28], ROBUST [19], and should be followed. More specifically, in the context of Bayesian borrowing in clinical trials, the recent FDA draft guidance [56] specifies in some detail documenting plans for Bayesian analyses. Moreover, we provide in Table 4 a summary of the key reporting gaps identified in this review and highlight the most important items that future studies planning to incorporate Bayesian borrowing should report.

**Table 4:** Summary of the key reporting gaps identified in this review and recommendations on crucial reporting items

Key reporting gaps identified	Recommended reporting for future Bayesian borrowing studies
Unclear source of borrowed data	Data sources explicitly stated (with accompanying details and with citations when applicable).
Insufficient details on the assessment of external data suitability	Comprehensive details on the external data relevance. Provide details on the similarity of patient populations, whether treatment evaluation was the same etc.
Not explicitly specifying the prior parametrization	Report the prior parametrization e.g. Beta(a,b). Additionally, clarify the parameter role when ambiguity may exist, e.g., scale or rate for Gamma priors.
Unclear dynamic borrowing method	Describe the borrowing method used clearly and give relevant citations. Provide implementation details, including software packages used.
Poor reporting and justification of borrowing parameters	Explicitly state the borrowing parameters and provide clear rationale for how the choice was made.

## Supplementary information

Supplementary Appendix

Checklist

## Data Availability

Available on request.
