# Supplementary material for: Uptake of Bayesian robust information borrowing in Phase II clinical trials: a scoping review
Source: BMC Med Res Methodol. 2026 Nov 5;26:247. doi: 10.1186/s12874-026-03052-3 (PMC13761132; doi:10.1186/s12874-026-03052-3)
Supplement: Supplementary file 1 [file 12874_2026_3052_MOESM1_ESM.pdf]

Uptake of Bayesian robust information borrowing in Phase II clinical trials: a scoping review

Vivienn Weru *[1,2], Silvia Calderazzo *[1], Leonhard Held[3], Manuel Wiesenfarth[4], and Annette Kopp-Schneider[1]

[1]Division of Biostatistics, German Cancer Research Center (DKFZ), Heidelberg, Germany
[2]Medical Faculty, Universität Heidelberg, Heidelberg, Germany
[3]Epidemiology, Biostatistics & Prevention Institute (EBPI), University of Zurich, Zurich, Switzerland
[4]Cogitars GmbH, Heidelberg, Germany

Supplementary Appendix

Contents

A Literature search **2**

B Key aspects on borrowing external data **6**
- B.1 Source of borrowed data 6
- B.2 Target of borrowing 6
- B.3 Assessment of external data suitability 7
- B.4 Borrowing methods 7
- B.5 Effective sample size 11
- B.6 Essential items to report in Bayesian analysis 12

C List of included articles **13**

D List of additional articles **18**

*Shared first author.

A Literature search

Table A.1: Our search was conducted in PubMed central which allows search in full text. We decided to search in the full text so as not to miss most clinical trials that would not have the key Bayesian borrowing keywords in the title or abstract. As our systematic review focuses on actual clinical trials using Bayesian borrowing, articles in statistical methodological journals were excluded.

1	“Bayesian” [all fields]
2	“Informed consent” OR “documented consent” [all fields]
3	”historical data” OR ”external data” OR ”dynamic borrowing” OR ”informative prior” OR ”informative priors” OR ”non-concurrent control” OR ”non-concurrent controls” OR ”historical control” OR ”historical controls” OR “historical information” OR “historical placebo” OR “informative previous distribution” OR “informative previous distributions” OR “exchangeability” OR ”evidence synthesis” OR “Bayesian borrowing” OR “hybrid control” OR “hybrid controls” [all fields]
4	”phase-II” OR ”phase II” OR ”proof-of-concept” OR ”proof of concept” OR ”early phase” OR ”safety and efficacy” OR “efficacy and safety” OR ”phase-2” OR ”phase 2a” OR “phase 2b” OR “phase 2” [all fields]
5	”Pharmaceutical statistics” OR ”Biometrics” OR ” Biometrical journal” OR ”Statistics in Medicine” OR ”Statistical Methods in Medical Research” OR ”Journal of Biopharmaceutical Statistics” OR ”Statistics in Biopharmaceutical Research” OR ”Research Synthesis Methods” OR ”Biostatistics” OR ”Bayesian Analysis” OR ”Computer Methods and Programs in Biomedicine” OR ”arXiv” OR ”Canadian Journal of Statistics” OR ”Computational Statistics & Data Analysis” OR ”Journal of the Royal Statistical Society” OR ”Statistics in Biosciences” OR ”Statistical science” OR ”medRxiv” [journal]
6	“Posters” OR “abstracts” OR “abstract” OR “abstract book” OR “poster presentations” OR “Annual meeting” OR “conference abstracts” OR “pre-conference program*” OR “poster session” OR “oral presentations” OR “abstract presentations” OR “conference” OR “congress” OR “scientific programme” OR “symposium” OR “systematic review” OR “literature review” OR “scoping review” OR “narrative review” OR “: a review” OR “—a review” OR “survey” [title]
Search equation	#1 AND #2 AND #3 AND #4 NOT #5 NOT #6

Table A.2: We additionally included the following search in Cochrane Central Register of Controlled Trials

1	"Bayesian" [all text field]
2	"historical data" OR "external data" OR "dynamic borrowing" OR "informative prior" OR "non-concurrent control" OR "historical control" OR "historical information" OR "historical placebo" OR "informative previous distribution" OR "exchangeability" OR "evidence synthesis" OR "Bayesian borrowing" OR "hybrid control" [all text field]
3	"phase-II" OR "phase II" OR "proof-of-concept" OR "proof of concept" OR "early phase" OR "safety and efficacy" OR "efficacy and safety" OR "phase-2" OR "phase 2a" OR "phase 2b" OR "phase 2" [all text field]
Search equation	#1 AND #2 AND #3

Table A.3: List of statistical methodological papers for which citation searching was done.

- Schmidli, H. et al. "Robust meta-analytic-predictive priors in clinical trials with historical control information". In: *Biometrics* 70.4 (2014), pp. 1023–1032. DOI: 10.1111/biom.12242
- Ibrahim, J. G. et al. "Power prior distributions for regression models". In: *Statistical Science* (2000), pp. 46–60. DOI: 10.1214/ss/1009212673
- Ibrahim, J. G. et al. "The power prior: theory and applications". In: *Statistics in Medicine* 34.28 (2015), pp. 3724–3749. DOI: 10.1002/sim.6728
- Neuenschwander, B. et al. "Summarizing historical information on controls in clinical trials". In: *Clinical Trials* 7.1 (2010), pp. 5–18. DOI: 10.1177/1740774509356002
- Neuenschwander, B. et al. "Use of Historical Data". In: *Bayesian Methods in Pharmaceutical Research*. Chapman and Hall/CRC, Apr. 2020, pp. 111–137. ISBN: 9781315180212. DOI: 10.1201/9781315180212-6. URL: http://dx.doi.org/10.1201/9781315180212-6
- Viele, K. et al. "Use of historical control data for assessing treatment effects in clinical trials". In: *Pharmaceutical Statistics* 13.1 (2014), pp. 41–54. DOI: 10.1002/pst.1589
- Berry, S. M. et al. "Bayesian hierarchical modeling of patient subpopulations: efficient designs of phase II oncology clinical trials". In: *Clinical Trials* 10.5 (2013), pp. 720–734. DOI: 10.1177/1740774513497539
- Thall, P. F. et al. "Hierarchical Bayesian approaches to phase II trials in diseases with multiple subtypes". In: *Statistics in Medicine* 22.5 (2003), pp. 763–780. DOI: 10.1002/sim.1399
- Neuenschwander, B. et al. "Robust exchangeability designs for early phase clinical trials with multiple strata". In: *Pharmaceutical Statistics* 15.2 (2016), pp. 123–134. DOI: 10.1002/pst.1730
- Hobbs, B. P. et al. "Commensurate priors for incorporating historical information in clinical trials using general and generalized linear models". In: *Bayesian Analysis (Online)* 7.3 (2012), p. 639. DOI: 10.1214/12-ba722
- Gravestock, I. et al. "Adaptive power priors with empirical Bayes for clinical trials". In: *Pharmaceutical Statistics* 16.5 (2017), pp. 349–360. DOI: 10.1002/pst.1814
- Weber, S. et al. "Applying meta-analytic-predictive priors with the R Bayesian evidence synthesis tools". In: *arXiv preprint arXiv:1907.00603* (2019). DOI: 10.18637/jss.v100.i19
- Lim, J. et al. "Minimizing patient burden through the use of historical subject-level data in innovative confirmatory clinical trials: review of methods and opportunities". In: *Therapeutic Innovation & Regulatory Science* 52.5 (2018), pp. 546–559. DOI: 10.1177/2168479018778282
- Hobbs, B. P. et al. "Bayesian basket trial design with exchangeability monitoring". In: *Statistics in Medicine* 37.25 (July 2018), pp. 3557–3572. ISSN: 1097-0258. DOI: 10.1002/sim.7893. URL: http://dx.doi.org/10.1002/sim.7893
- Jin, J. et al. "Bayesian methods for the analysis of early-phase oncology basket trials with information borrowing across cancer types". In: *Statistics in Medicine* 39.25 (July 2020), pp. 3459–3475. ISSN: 1097-0258. DOI: 10.1002/sim.8675. URL: http://dx.doi.org/10.1002/sim.8675
- Chu, Y. et al. "A Bayesian basket trial design using a calibrated Bayesian hierarchical model". In: *Clinical Trials* 15.2 (Mar. 2018), pp. 149–158. ISSN: 1740-7753. DOI: 10.1177/1740774518755122. URL: http://dx.doi.org/10.1177/1740774518755122
- Psioda, M. A. et al. "Bayesian adaptive basket trial design using model averaging". In: *Biostatistics* 22.1 (May 2019), pp. 19–34. ISSN: 1468-4357. DOI: 10.1093/biostatistics/kxz014. URL: http://dx.doi.org/10.1093/biostatistics/kxz014
- Neuenschwander, B. et al. "On the Use of Co-Data in Clinical Trials". In: *Statistics in Biopharmaceutical Research* 8.3 (July 2016), pp. 345–354. ISSN: 1946-6315. DOI: 10.1080/19466315.2016.1174149. URL: http://dx.doi.org/10.1080/19466315.2016.1174149

Table A.4: Data extracted from studies

- Study ID
- Title of the article
- Publication year
- Was the article a protocol or report?
- What was the intervention type (Drug, Medical device, surgical, etc)?
- What was the disease area? (Oncology, Infectious Diseases etc)
- If Oncology, what type of cancer?
- Was the study a master protocol?
- If master protocol, specify which type
- Was the statistical model specified?
- Was the analytical technique specified? (e.g. MCMC)
- Was the prior distribution specified?
- Was the prior distribution justified?
- To which did they borrow? (control arm, treatment effect..)
- What was the source of the external data? (previous trials, real-world evidence, expert opinion..)
- Did they explicitly cite the source of the external data?
- Was this included in the protocol?
- For those that used expert opinion elicitation, did they use a formal framework e.g. SHELF?
- How was similarity between historical and current studies assessed?
- Which borrowing method did they use?
- For those that used fixed downweighting, how was the discounting weight arrived at?
- For those that used Robust MAP, what weight was assigned to the informative component?
- How was this weight decided upon?
- For the trials that used the robust MAP, how vague was the robust component?
- For the trials that used the robust MAP with normal endpoint, where was the robust component located?
- For the trials that used the MAP prior, what prior was used for the between study heterogeneity parameter?
- For trials that used Bayesian hierarchical model, what prior was used for the heterogeneity parameter?
- For those that used commensurate prior, what prior was used for the commensurability parameter?
- Was historical data reported in terms of Effective sample size?
- Which ESS method did they use?
- What was the ratio of historical effective sample size to current data sample size?
- Was a sensitivity analysis conducted in addition, to check the influence of the borrowing parameters on the results?
- Was a central tendency measure reported?
- Did they report standard deviation or credible intervals?
- Which success criterion did they use?

B Key aspects on borrowing external data

B.1 Source of borrowed data

In borrowing data, the sources of borrowing can be external or internal. External sources include previous trials, Real-World evidence (RWE) or information elicited from experts. RWE refers to data obtained outside the confines of a strictly controlled trial, *e.g.*, patient registries [8]. In elicitation from experts, the SHeffield ELicitation Framework (SHELF) [9] is a commonly used structured approach for experts to express opinions through probability distributions which can then be used as informative priors. Morgan et al. [10] have conducted a systematic review identifying methods of expert elicitation used in clinical trials. Internal sources include borrowing between arms within a trial, for example, in basket trials. Basket trials are a specific type of a clinical trial where a single therapy is evaluated across different types of tumors with a common mutation (see *e.g.* [11–13]). In such a setting, especially if some arms might have small sample sizes, information is borrowed across arms. Platform trials represent a special case of borrowing within a trial. These trials involve a shared infrastructure allowing multiple treatment arms to be compared to a common control group, with such interventions able to enter or leave the trial over time (see *e.g.* [13–15] for more information on platform trials).

Distinguishing between different sources of external data is important as different assumptions, for example when evaluating operating characteristics, may be applicable depending on the source of data. Information obtained from historical trials or elicited from experts would be known at the design stage. For concurrent and same trial information, however, the information is obtained alongside the current trial and, therefore, not known at the design stage. With respect to RWE, while such data may be available, it may not have been extracted yet.

B.2 Target of borrowing

External information may be borrowed for the control arm of a study: a clinical trial investigating efficacy of a novel treatment compared to the standard of care may use the information available on the standard of care from previous studies. Borrowing external information to the control arm leads to the so-called hybrid-control designs. As there is usually quite some information available on the control/standard of care arm, such designs have seen increased usage [16, 17]. Other times the external information available may be on the treatment effect directly, for example, as is the case in one arm trials, or when information is summarized in terms of treatment effect. In basket trials, as there is typically no control arm, information is borrowed between the treatment arms of the different baskets.

B.3 Assessment of external data suitability

As mentioned before, the external data needs careful selection. Pocock [18] outlined six conditions for historical and current controls comparability:

1. The group must have been treated with exactly the same treatment as the randomized controls in the current study.
2. The group must have been part of a recent clinical study with the same requirements for patient eligibility.
3. The methods of treatment evaluation must be the same.
4. The distributions of important patient characteristics in the group should be comparable with those in the new trial.
5. The historical study must have been performed in the same organization with largely the same clinical investigators.
6. There must be no other indications leading one to expect different results between the current and the historical controls.

In this review, we evaluated whether the studies assessed Pocock's criteria. One may also adjust for relevant covariates in the analysis, though this requires access to the patient-level data.

B.4 Borrowing methods

Even if the external data has been careful selected, there might still be differences between the current and external information. To mitigate risks associated with prior-data conflict, different methods have been proposed for incorporating the external data. A description of the main borrowing methods is provided below.

Power prior The power prior approach [2, 3] assumes a common parameter for the external and current data, but downweights the likelihood of the external data. Suppose D_{ext} represents the external data generated by θ_{ext}. Assuming an initial vague prior π_0, the posterior distribution for the external data parameter is obtained as

$$\pi(\theta_{ext} \mid D_{ext}) \propto L(D_{ext} \mid \theta_{ext})\pi_0(\theta_{ext}).$$

With interest in the inference of parameter θ for the current data, the power prior assumes $\theta_{ext} = \theta$, but downweights the external data to fulfill such an assumption. The posterior distribution above would then form the prior for the current data with the likelihood of the external data downweighted.

The power prior for the current parameter θ would then be constructed as follows;

$$\pi(\theta \mid \alpha, D_{ext}) \propto L(D_{ext} \mid \theta)^{\alpha} \pi_0(\theta),$$

where α, $0 \leq \alpha \leq 1$, is the power parameter. The downweighting is, therefore, driven by α, with $\alpha = 0$ corresponding to no inclusion of the external data and $\alpha = 1$ corresponding to pooling the current and external data. Different ways have been proposed for obtaining the value of α. All publications identified used a fixed value of α apriori (aka conditional power prior), for instance by eliciting its value from experts, in which case borrowing does not adapt to drift and is not dynamic. Dynamic approaches could be used, however. Gravestock et al. [7] propose an empirical Bayes approach where α is estimated based on the data. A different approach from the above assigns a prior for the power parameter (See e.g. Neuenschwander et al. [19] and Banbeta et al. [20]). Choice of α, therefore, needs to be reported and justification for the choice provided.

Meta-analytic predictive (MAP) prior Another dynamic borrowing approach is the meta-analytic predictive (MAP) prior proposed by Neuenschwander et al. [4]. Suppose there are K external studies each with data D_k modeled by $f(D_k \mid \theta_k)$, where θ_k is the corresponding parameter. Suppose further that θ is the current data parameter. The meta-analytic predictive approach assumes the model parameters of the external and current data to be exchangeable. Specifically,

$$\theta_1, \ldots, \theta_K, \theta \sim N(\mu, \tau^2),$$

where μ is the population mean and τ^2 is the between-trial heterogeneity parameter. The MAP prior $\pi_{MAP}(\theta) = \pi(\theta \mid D_1, \ldots D_K)$. The heterogeneity parameter above drives the borrowing; a small value is akin to pooling the external and current data, while a large value corresponds to independent analysis where the external data plays no role. A fully Bayesian approach is often take with a non-informative prior used for μ while an informative prior is typically given to τ^2. The half-t prior distributions have been recommended for τ [21], especially when the number of external studies is small. The inverse-gamma (α, β) prior has been investigated in the literature as a potential alternative prior for τ^2 but has been shown to be very sensitive to the input parameters α and β [21, 22]. While the MAP prior was initially designed for multiple external studies setting, Röver et al. [23] illustrate the MAP prior based on a single study. Derivation of the MAP prior under non-conjugate prior choices involves MCMC and a translation to a parametric form is needed. To ease the computational and communication burden associated with this, it has been proposed to approximate the MAP prior by a mixture of conjugate priors [1, 24, 25].

Robust MAP prior To protect against potential prior-data conflict, Schmidli et al. [1] proposes to "robustify" the MAP prior above. Specifically, the robust MAP prior is given as follows;

$$\pi_{RMAP}(\theta) = w\pi_{MAP}(\theta) + (1-w)\pi_{robust}(\theta), \quad (1)$$

where w is the prior weight assigned to the MAP prior and $\pi_{robust}(\theta)$ is the robustifying component. The robust MAP above is combined with the current data D, to yield a posterior distribution of the form

$$\pi_{\text{RMAP}}(\theta \mid D) = \tilde{w}\pi_{\text{MAP}}(\theta \mid D) + (1-\tilde{w})\pi_{\text{robust}}(\theta \mid D). \quad (2)$$

Dynamic borrowing is then achieved via update of the weight, where the weight is updated as follows;

$$\tilde{w} = \frac{w m_{\text{MAP}}(D)}{w m_{\text{MAP}}(D) + (1-w) m_{\text{robust}}(D)},$$

where $m_{\text{MAP}}(D)$ and $m_{\text{robust}}(D)$ are the marginal likelihoods of the data under the MAP and robust component, respectively.
From (1), it is clear that in addition to formulation of the MAP prior, the prior weight w as well as the robust component need to be chosen. The prior weight can be elicited from experts, for example using a dedicated elicitation framework like the Sheffield Elicitation Framework (SHELF) [9]. The choice of $\pi_{robust}(\theta)$ is also crucial. Weru et al. [26] investigate the choice of parameters for the robust mixture prior, illustrating how some choices can lead to undesired performances.

Bayesian hierarchical model (BHM) In basket trials, the Bayesian hierarchical Model (BHM) is the historically most well-known approach used to facilitate borrowing in this context [5, 6]. Suppose there is a basket trial with K baskets/cohorts each of size n_k. We consider a binary endpoint since these are common in basket trials. Suppose y_k is the number of responders in basket k, and

$$y_k \sim Bin(n_k, \pi_k),$$

where π_k is the true response rate in basket k. A logit transformation of π_k is commonly used, here denoted as $\theta_k = \log(\frac{\pi_k}{1-\pi_k})$. In this model, $\theta_k \sim N(\mu, \tau^2)$, where μ is the population mean and τ^2 is the between-cohorts variance. The degree of borrowing is determined by τ, with 0 corresponding to pooling and $\tau \to \infty$ corresponding to independent analysis. Again, the choice of τ^2 is, therefore, crucial. Similar considerations as in the MAP prior above for τ^2 apply.

Clustered BHMs (CBHM) have also been suggested that group similar cohorts into subgroups such that information is borrowed depending on the similarity within subgroups. In this context, Chen et al. [27] propose using a Dirichlet process to dynamically determine the number of clusters.

Fujikawa's approach Fujikawa et al. [28] proposed another approach to share information across baskets. The approach proceeds in two steps. In the first step, the baskets are analyzed individually to obtain the basket-specific posterior distributions. Suppose there are r_k observed responders in basket k of size n_k with a true response probability p_k. Given a beta prior distribution, $Beta(a_k, b_k)$, for p_k, the resulting posterior distribution in basket k is given as follows;

$$\pi(p_k \mid D_k) = Beta(a_k + r_k, b_k + n_k - r_k),$$

where $D_k = \{r_k, n_k\}$ is the data for basket k. In the second step, the final posterior distribution for basket k is obtained where information is now shared between baskets. Specifically, the posterior distribution for basket k in this step is a weighted sum of the individual posterior parameters as follows;

$$\pi(p_k \mid \mathbf{D}) = Beta\left(\sum_{i=1}^{K} w_{k,i}(a_i + r_i), \sum_{i=1}^{K} w_{k,i}(b_i + n_i - r_i)\right).$$

The weights measure similarity between the posterior distributions in step 1 for two baskets. The authors use the Jensen-Shannon divergence (JSD) to compute the weights as follows;

$$w_{k,i} = \begin{cases} (1 - \text{JSD}(\pi(p_k \mid D_k), \pi(p_i \mid D_i))^{\epsilon} & \text{if} \quad (1 - \text{JSD}(\pi(p_k \mid D_k), \pi(p_i \mid D_i))^{\epsilon} > \zeta, \\ 0 & \text{otherwise,} \end{cases}$$

where ϵ and ζ are tuning parameters. These need to be specified and justified.

Adjusting for covariates, e.g. through linear regression or matching approaches, to correct for measured confounding between the current and external data can also be used. Nonetheless, the dynamic borrowing methods above help remedy problems associated with unmeasured confounding.

A summary of the tuning parameters that require specification and justification for the different borrowing methods is provided in Table B.5 below.

Table B.5: Summary of the tuning parameters to be justified for the borrowing methods identified in the review. Depending on the method, tuning parameters may be fixed or may be assumed to follow a prior (the distribution of which and hyperparameters to be selected). In contrast to the other methods, the fixed power prior fixes the strength of borrowing independent of the drift.

Borrowing method	Tuning parameter
Fixed power prior	α
Meta-analytic predictive (MAP) prior	τ^2
Robust MAP prior	w and $\pi_{robust}(\theta)$
Bayesian hierarchical model (BHM)	τ
Fujikawa's approach	ϵ and ζ

All borrowing methods rely on parameter settings that regulate the amount of information borrowed.

B.5 Effective sample size

Quantifying the informativeness and impact of the prior is of key importance. The informative prior can be translated into a more intuitive metric called the prior effective sample size (ESS) which represents, in simple cases, how many observations the prior is worth. In simple settings, the ESS can be easily obtained. For example, for a binomial likelihood with a conjugate $Beta(a, b)$ prior, the ESS is easily obtained as $a + b$. For a normal prior with variance τ^2 for the mean of data from a normal distribution with variance σ^2, the ESS is simply σ^2/τ^2 [29]. For more complex settings, different approaches have been suggested for calculating the ESS of a prior, with computations becoming non-trivial for conjugate mixture priors and non-conjugate priors. A description of the main ESS methods is provided below.

Suppose the prior of interest is denoted as $\pi(\theta)$. Such a prior can be thought of as the posterior of an earlier study, denoted as $q_m(\theta \mid D_{ext})$, where a vague prior was combined with the data D_{ext} of sample size m. The approach by Morita et al. [30] involves obtaining the sample size m by matching the curvature of these two densities at the mean.

Hobbs et al. [31] propose a computation of the ESS based on the ratio of posterior precision as follows;

$$n * \left\{ \frac{prec(\theta \mid D, D_{ext})}{prec(\theta \mid D)} - 1 \right\},$$

where $prec(\theta \mid D, D_{ext})$ is the posterior precision of θ under the model incorporating external data, $prec(\theta \mid D)$ is the posterior precision of θ under a reference prior and n is the current data sample size.

Neuenschwander et al. [32] propose the so-called expected local-information-ratio (ELIR) ESS. This ESS is a ratio of the information of the prior of interest and the expected Fisher information of one information unit, integrated over the prior distribution of θ. Specifically,

$$ESS_{\mathrm{ELIR}} = E_\theta \left\{ \frac{i(\pi(\theta))}{i_F(\theta)} \right\},$$

where $i_F(\theta) = -E_{D_1 \,|\, \theta}\{\frac{d^2 log f(D_1 \,|\, \theta))}{d\theta^2}\}$ and $i(\pi(\theta)) = -\frac{d^2 log \pi(\theta)}{d\theta^2}$.

The above ESS metrics are, however, independent of the current data. Wiesenfarth et al. [33] propose the effective current sample size (ECSS) that takes into account potential prior-data conflict. They consider a posterior arising from combining a reference prior with data of sample size k, and a posterior combining the prior of interest and data of sample size $k-m$. The aim is then to find the m that minimizes the difference in the MSE induced by the posterior mean of the two posteriors,

$$MSE\{\pi(\theta \,|\, D_{1:(k-m)})\} - MSE\{\pi_{reference}(\theta \,|\, D_{1:k})\}.$$

B.6 Essential items to report in Bayesian analysis

Clear reporting of Bayesian analysis, as with any statistical analysis, is crucial to ensure understanding and transparency of the analysis to readers. When an informative prior is used, clear explanation of the choice of prior is needed. To aid in standardized reporting of Bayesian analyses, several guidelines have been developed. Sung et al. [34] developed the so-called ROBUST (Reporting Of Bayes Used in clinical STudies) items. ROBUST is a list of seven items deemed important in reporting Bayesian analysis of a clinical trial, and grouped into three categories pertaining to the prior distribution, analysis and results. Spiegelhalter et al. [35] proposed a 10-item checklist (BayesWatch) in the context of health technology assessment detailing key items to report in a Bayesian analysis to ensure replicability of the analysis. In addition to the usual items like prior distribution specification, BayesWatch asks for items such as clear description of the study design as well as the loss function used. Most recently, Kruschke [36] proposed BARG (Bayesian Analysis Reporting guidelines), which is a more extensive and general guideline on reporting of Bayesian analysis, and largely subsumes ROBUST and BayesWatch.

C List of included articles

1. Richeldi, L. et al. “Trial of a preferential phosphodiesterase 4B inhibitor for idiopathic pulmonary fibrosis”. In: *New England Journal of Medicine* 386.23 (2022), pp. 2178–2187. DOI: 10.1056/nejmoa2201737

2. Hueber, W. et al. “Secukinumab, a human anti-IL-17A monoclonal antibody, for moderate to severe Crohn’s disease: unexpected results of a randomised, double-blind placebo-controlled trial”. In: *Gut* 61.12 (2012), pp. 1693–1700. DOI: 10.1136/gutjnl-2011-301668

3. Pozzilli, P. et al. “Randomized 52-week phase 2 trial of albiglutide versus placebo in adult patients with newly diagnosed type 1 diabetes”. In: *The Journal of Clinical Endocrinology & Metabolism* 105.6 (2020), e2192–e2206. DOI: 10.1210/clinem/dgaa149

4. Amuge, P. et al. “Once-daily dolutegravir-based antiretroviral therapy in infants and children living with HIV from age 4 weeks: results from the below 14 kg cohort in the randomised ODYSSEY trial”. In: *The Lancet HIV* 9.9 (2022), e638–e648. DOI: 10.1016/S2352-3018(22)00163-1

5. Maertens, J. et al. “Phase 2 study of anti-human cytomegalovirus monoclonal antibodies for prophylaxis in hematopoietic cell transplantation”. In: *Antimicrobial Agents and Chemotherapy* 64.4 (2020), pp. 10–1128. DOI: 10.1128/aac.02467-19

6. Baeten, D. et al. “Anti-interleukin-17A monoclonal antibody secukinumab in treatment of ankylosing spondylitis: a randomised, double-blind, placebo-controlled trial”. In: *The Lancet* 382.9906 (2013), pp. 1705–1713. DOI: 10.1016/s0140-6736(13)61134-4

7. Glatt, S. et al. “Efficacy and safety of bimekizumab as add-on therapy for rheumatoid arthritis in patients with inadequate response to certolizumab pegol: a proof-of-concept study”. In: *Annals of the Rheumatic Diseases* 78.8 (2019), pp. 1033–1040. DOI: 10.1136/annrheumdis-2018-214943

8. Fanale, M. et al. “Phase IA/II, multicentre, open-label study of the CD 40 antagonistic monoclonal antibody lucatumumab in adult patients with advanced non-Hodgkin or Hodgkin lymphoma”. In: *British Journal of Haematology* 164.2 (2014), pp. 258–265. DOI: 10.1111/bjh.12630

9. Fels, L. M. et al. “The effect of a combined indomethacin and levonorgestrel-releasing intrauterine system on short-term postplacement bleeding profile: a randomized proof-of-concept trial”. In: *American Journal

of Obstetrics and Gynecology 228.3 (2023), 322–e1. DOI: 10.1016/j.ajog.2022.10.025

10. Frenel, J.-S. et al. "Durvalumab and tremelimumab in combination with metronomic oral vinorelbine for recurrent advanced cervical cancer: an open-label phase I/II study". In: *Journal for Immunotherapy of Cancer* 13.1 (2025), e010708. DOI: 10.1136/jitc-2024-010708

11. Gale, J. D. et al. "Effect of PF-04634817, an oral CCR2/5 chemokine receptor antagonist, on albuminuria in adults with overt diabetic nephropathy". In: *Kidney International Reports* 3.6 (2018), pp. 1316–1327. DOI: 10.1016/j.ekir.2018.07.010

12. Glatt, S. et al. "Efficacy and safety of bimekizumab in moderate to severe hidradenitis suppurativa: a phase 2, double-blind, placebo-controlled randomized clinical trial". In: *JAMA Dermatology* 157.11 (2021), pp. 1279–1288. DOI: 10.1001/jamadermatol.2021.2905

13. Radeke, H. H. et al. "A multicentre, double-blind, placebo-controlled, parallel-group study to evaluate the efficacy, safety, and tolerability of the S1P receptor agonist KRP203 in patients with moderately active refractory ulcerative colitis". In: *Inflammatory Intestinal Diseases* 5.4 (2020), pp. 180–190. DOI: 10.1159/000509393

14. ATTACC, A.-4. et al. "Therapeutic anticoagulation with heparin in noncritically ill patients with Covid-19". In: *New England Journal of Medicine* 385.9 (2021), pp. 790–802. DOI: 10.1056/nejmoa2105911

15. Piha-Paul, S. A. et al. "Efficacy and safety of buparlisib, a PI3K inhibitor, in patients with malignancies harboring a PI3K pathway activation: a phase 2, open-label, single-arm study". In: *Oncotarget* 10.60 (2019), p. 6526. DOI: 10.18632/oncotarget.27251

16. Poonai, N. et al. "Adaptive randomised controlled non-inferiority multicentre trial (the Ketodex Trial) on intranasal dexmedetomidine plus ketamine for procedural sedation in children: study protocol". In: *BMJ open* 10.12 (2020), e041319. DOI: 10.1136/bmjopen-2020-041319

17. Jones, R. L. et al. "A phase 1 and randomized controlled phase 2 trial of the safety and efficacy of the combination of gemcitabine and docetaxel with ontuximab (MORAb-004) in metastatic soft-tissue sarcomas". In: *Cancer* 125.14 (2019), pp. 2445–2454. DOI: 10.1002/cncr.32084

18. Kawamoto, Y. et al. "Phase II trial of niraparib for BRCA-mutated biliary tract, pancreatic and other gastrointestinal cancers: NIR-B".

in: *Future Oncology* 20.26 (2024), pp. 1901–1907. DOI: 10.2217/fon-2023-0348

19. Ludbrook, V. J. et al. "Inhibition of receptor-interacting protein kinase 1 in chronic plaque psoriasis: a multicenter, randomized, double-blind, placebo-controlled study". In: *Dermatology and Therapy* 14.2 (2024), pp. 489–504. DOI: 10.1007/s13555-024-01097-0

20. Goligher, E. C. et al. "Therapeutic Anticoagulation with Heparin in Critically Ill Patients with Covid-19". In: *The New England Journal of Medicine* (2021). URL: https://api.semanticscholar.org/CorpusID:236926251

21. Brandes, A. A. et al. "A Phase II randomized study of galunisertib monotherapy or galunisertib plus lomustine compared with lomustine monotherapy in patients with recurrent glioblastoma". In: *Neuro-oncology* 18.8 (2016), pp. 1146–1156. DOI: 10.1093/neuonc/now009

22. Brard, C. et al. "Sarcome-13/OS2016 trial protocol: a multicentre, randomised, open-label, phase II trial of mifamurtide combined with postoperative chemotherapy for patients with newly diagnosed high-risk osteosarcoma". In: *BMJ Open* 9.5 (2019), e025877. DOI: 10.1136/bmjopen-2018-025877

23. Chugh, R. et al. "Phase II multicenter trial of imatinib in 10 histologic subtypes of sarcoma using a Bayesian hierarchical statistical model". In: *Journal of Clinical Oncology* 27.19 (2009), pp. 3148–3153. DOI: 10.1200/jco.2008.20.5054

24. McIlroy, G. et al. "Investigator choice of standard therapy versus sequential novel therapy arms in the treatment of relapsed follicular lymphoma (REFRACT): study protocol for a multi-centre, open-label, randomised, phase II platform trial". In: *BMC Cancer* 24.1 (2024), p. 370. DOI: 10.1186/s12885-024-12112-0

25. Melisi, D. et al. "Galunisertib plus gemcitabine vs. gemcitabine for first-line treatment of patients with unresectable pancreatic cancer". In: *British Journal of Cancer* 119.10 (2018), pp. 1208–1214. DOI: 10.1038/s41416-018-0246-z

26. Muehlemann, N. et al. "Innovative adaptive study design in transfusion-dependent beta-thalassemia: Bayesian design with concurrent randomization and borrowing from historical data". In: *Blood* 138 (2021), p. 4160. DOI: 10.1182/blood-2021-146512

27. Olson, A. et al. "Third-party BK virus-specific cytotoxic T lymphocyte therapy for hemorrhagic cystitis following allotransplantation". In:

Journal of Clinical Oncology 39.24 (2021), pp. 2710–2719. DOI: 10.1200/jco.20.02608

28. Robinson, M. F. et al. "Efficacy and safety of PF-06651600 (Ritlecitinib), a novel Jak3/TEC inhibitor, in patients with Moderate-To-Severe rheumatoid arthritis and an inadequate response to methotrexate". In: *Arthritis & Rheumatology* 72.10 (2020), pp. 1621–1631. DOI: 10.1002/art.41316

29. Salloway, S. et al. "A trial of gantenerumab or solanezumab in dominantly inherited Alzheimer's disease". In: *Nature medicine* 27.7 (2021), pp. 1187–1196. DOI: 10.1038/s41591-021-01369-8

30. Scheele, W. et al. "Phosphodiesterase type 5 inhibition reduces albuminuria in subjects with overt diabetic nephropathy". In: *Journal of the American Society of Nephrology* 27.11 (2016), pp. 3459–3468. DOI: 10.1681/asn.2015050473

31. Schuetze, S. M. et al. "SARC009: phase 2 study of dasatinib in patients with previously treated, high-grade, advanced sarcoma". In: *Cancer* 122.6 (2016), pp. 868–874. DOI: 10.1002/cncr.29858

32. Schuler, M. et al. "Nivolumab in patients with advanced or metastatic malignancies, including rare cancers: results of checkmate 627, an adaptive basket design clinical trial". In: *JCO Oncology Advances* 1 (2024), e2400044. DOI: 10.1200/oa-24-00044

33. Seccia, T. M. et al. "Randomized clinical trial of nafamostat mesylate, a potent transmembrane protease serine 2 (TMPRSS2) inhibitor, in patients with COVID-19 pneumonia". In: *Journal of Clinical Medicine* 12.20 (2023), p. 6618. DOI: 10.3390/jcm12206618

34. Slosberg, E. D. et al. "Signature program: a platform of basket trials". In: *Oncotarget* 9.30 (2018), p. 21383. DOI: 10.18632/oncotarget.25109

35. Subbiah, V. et al. "Dabrafenib plus trametinib in patients with BRAF V600E-mutant anaplastic thyroid cancer: updated analysis from the phase II ROAR basket study". In: *Annals of Oncology* 33.4 (2022), pp. 406–415. DOI: 10.1016/j.annonc.2021.12.014

36. Taylor, M. H. et al. "A mutation-specific, single-arm, phase 2 study of dovitinib in patients with advanced malignancies". In: *Oncotarget* 11.14 (Apr. 2020), pp. 1235–1243. ISSN: 1949-2553. DOI: 10.18632/oncotarget.27530. URL: http://dx.doi.org/10.18632/oncotarget.27530

37. Visvanathan, S. et al. "Effects of BI 655064, an antagonistic anti-CD40 antibody, on clinical and biomarker variables in patients with active rheumatoid arthritis: a randomised, double-blind, placebo-controlled, phase IIa study". In: *Annals of the Rheumatic Diseases* 78.6 (2019), pp. 754–760. DOI: 10.1136/annrheumdis-2018-214729

D List of additional articles

1. Rasschaert, G. et al. "AZUR-4, a Phase 2, Open Label, Randomized Study of Neoadjuvant Dostarlimab Plus CAPEOX Versus CAPEOX in Previously Untreated T4N0 or Stage III Mismatch Repair Proficient/Microsatellite Stable Resectable Colon Cancer". In: *Clinical Colorectal Cancer* 25.2 (2026), 302–307.e3. ISSN: 1533-0028. DOI: 10.1016/j.clcc.2026.03.001. URL: http://dx.doi.org/10.1016/j.clcc.2026.03.001

2. Weitz, J. I. et al. "Efficacy and safety of REGN9933A2 and REGN7508Cat for preventing postoperative venous thromboembolism (ROXI-VTE-I and ROXI-VTE-II): two randomised, open-label, phase 2 trials". In: *The Lancet* 406.10519 (Nov. 2025), pp. 2551–2563. ISSN: 0140-6736. DOI: 10.1016/s0140-6736(25)02097-5. URL: http://dx.doi.org/10.1016/s0140-6736(25)02097-5

3. Forman, S. et al. "A randomized, double-blind, placebo-controlled phase 2 study of eltrekibart, a novel septa-specific monoclonal antibody to CXCR1/2 ligands, in adults with hidradenitis suppurativa". In: *Journal of the American Academy of Dermatology* 94.2 (Feb. 2026), pp. 530–538. ISSN: 0190-9622. DOI: 10.1016/j.jaad.2025.10.015. URL: http://dx.doi.org/10.1016/j.jaad.2025.10.015

4. Yee, C.-S. et al. "EFAVALEUKIN ALFA IN PATIENTS WITH ACTIVE SYSTEMIC LUPUS ERYTHEMATOSUS: RESULTS OF A BAYESIAN ADAPTIVE, PHASE 2B, DOUBLE-BLIND, RANDOMIZED, PLACEBO-CONTROLLED DOSE-RANGING STUDY". in: *The Journal of Rheumatology* 52.Suppl 1 (May 2025), pp. 253.1–253. ISSN: 1499-2752. DOI: 10.3899/jrheum.2025-0390.pv277. URL: http://dx.doi.org/10.3899/jrheum.2025-0390.pv277

5. Barbar-Smiley, F. et al. "ROZIBAFUSP ALFA IN PATIENTS WITH ACTIVE SYSTEMIC LUPUS ERYTHEMATOSUS: RESULTS OF A BAYESIAN ADAPTIVE PHASE 2B, RANDOMIZED, DOUBLE-BLIND, PLACEBO-CONTROLLED, DOSE-RANGING STUDY". in: *The Journal of Rheumatology* 52.Suppl 1 (May 2025), pp. 73–74. ISSN: 1499-2752. DOI: 10.3899/jrheum.2025-0390.pt014. URL: http://dx.doi.org/10.3899/jrheum.2025-0390.pt014

6. Stanton, L. et al. "Randomised Phase II study of acalabrutinib in combination with R-CHOP treatment for diffuse large B-cell lymphoma confirms safety and unimpaired treatment delivery". In: *Blood* 146.Supplement 1 (Nov. 2025), pp. 5459–5459. ISSN: 1528-0020. DOI: 10.1182/blood-2025-5459. URL: http://dx.doi.org/10.1182/blood-2025-5459

7. Peyrin-Biroulet, L. et al. “Efficacy and Safety of Ravagalimab in Patients with Moderate-to-Severe Ulcerative Colitis: Data from a Phase 2a Trial”. In: *Advances in Therapy* 43.6 (Apr. 2026), pp. 2611–2630. ISSN: 1865-8652. DOI: 10.1007/s12325-026-03517-3. URL: http://dx.doi.org/10.1007/s12325-026-03517-3

8. Wuyts, W. A. et al. “Design of a phase II randomised, double-blind, placebo-controlled, dose-finding trial of BI 1819479 in patients with idiopathic pulmonary fibrosis”. In: *ERJ Open Research* 12.2 (Nov. 2025), pp. 00973–2025. ISSN: 2312-0541. DOI: 10.1183/23120541.00973-2025. URL: http://dx.doi.org/10.1183/23120541.00973-2025

9. Maher, T. M. et al. “Deupirfenidone Compared with Pirfenidone and Placebo in Idiopathic Pulmonary Fibrosis (ELEVATE-IPF): a Phase 2b Randomized Placebo-Controlled Trial”. In: *American Journal of Respiratory and Critical Care Medicine* (Apr. 2026). ISSN: 1535-4970. DOI: 10.1093/ajrccm/aamag155. URL: http://dx.doi.org/10.1093/ajrccm/aamag155

References

[1] Schmidli, H. et al. “Robust meta-analytic-predictive priors in clinical trials with historical control information”. In: *Biometrics* 70.4 (2014), pp. 1023–1032. DOI: 10.1111/biom.12242.

[2] Ibrahim, J. G. and Chen, M.-H. “Power prior distributions for regression models”. In: *Statistical Science* (2000), pp. 46–60. DOI: 10.1214/ss/1009212673.

[3] Ibrahim, J. G. et al. “The power prior: theory and applications”. In: *Statistics in Medicine* 34.28 (2015), pp. 3724–3749. DOI: 10.1002/sim.6728.

[4] Neuenschwander, B. et al. “Summarizing historical information on controls in clinical trials”. In: *Clinical Trials* 7.1 (2010), pp. 5–18. DOI: 10.1177/1740774509356002.

[5] Berry, S. M. et al. “Bayesian hierarchical modeling of patient subpopulations: efficient designs of phase II oncology clinical trials”. In: *Clinical Trials* 10.5 (2013), pp. 720–734. DOI: 10.1177/1740774513497539.

[6] Thall, P. F. et al. “Hierarchical Bayesian approaches to phase II trials in diseases with multiple subtypes”. In: *Statistics in Medicine* 22.5 (2003), pp. 763–780. DOI: 10.1002/sim.1399.

[7] Gravestock, I., Held, L., and consortium, C.-N. “Adaptive power priors with empirical Bayes for clinical trials”. In: *Pharmaceutical Statistics* 16.5 (2017), pp. 349–360. DOI: 10.1002/pst.1814.

[8] Franklin, J. M. et al. “Evaluating the Use of Nonrandomized Real-World Data Analyses for Regulatory Decision Making”. In: *Clinical Pharmacology & Therapeutics* 105.4 (Feb. 2019), pp. 867–877. ISSN: 1532-6535. DOI: 10.1002/cpt.1351. URL: http://dx.doi.org/10.1002/cpt.1351.

[9] O’Hagan, A. and Oakley, J. “SHELF: the Sheffield elicitation framework”. In: *Reference Source* (2013).

[10] Morgan, L. M. et al. “Comparing Methods of Expert Elicitation for Treatment Effect or Borrowing Parameters in Standard and Rare Disease Clinical Trials: A Systematic Mapping Study”. In: *arXiv preprint arXiv:2508.06288* (2025). DOI: 10.48550/arXiv.2508.06288.

[11] Honap, S. et al. “Basket, umbrella, and platform trials: the potential for master protocol–based trials in inflammatory bowel disease”. In: *Gastroenterology* 167.4 (2024), pp. 636–642. DOI: 10.1053/j.gastro.2024.04.020.

[12] Hobbs, B. P. et al. “Basket trials: review of current practice and innovations for future trials”. In: *Journal of Clinical Oncology* 40.30 (2022), pp. 3520–3528. DOI: 10.1200/jco.21.02285.

[13] Park, J. J. H. et al. "Systematic review of basket trials, umbrella trials, and platform trials: a landscape analysis of master protocols". In: *Trials* 20.1 (2019), p. 572. DOI: 10.1186/s13063-019-3664-1.

[14] Roustit, M. et al. "Platform trials". In: *Therapies* 78.1 (2023), pp. 29–38. DOI: 10.1016/j.therap.2022.12.003.

[15] Burki, T. "Platform trials: the future of medical research?" In: *The Lancet Respiratory Medicine* 11.3 (2023), pp. 232–233. DOI: 10.1016/s2213-2600(23)00052-8.

[16] Ventz, S. et al. "The design and evaluation of hybrid controlled trials that leverage external data and randomization". In: *Nature Communications* 13.1 (Oct. 2022). ISSN: 2041-1723. DOI: 10.1038/s41467-022-33192-1. URL: http://dx.doi.org/10.1038/s41467-022-33192-1.

[17] Li, C. et al. "Hybrid-control arm construction using historical trial data for an early-phase, randomized controlled trial in metastatic colorectal cancer". In: *Communications Medicine* 2.1 (2022). ISSN: 2730-664X. DOI: 10.1038/s43856-022-00155-y. URL: http://dx.doi.org/10.1038/s43856-022-00155-y.

[18] Pocock, S. J. "The combination of randomized and historical controls in clinical trials". In: *Journal of Chronic Diseases* 29.3 (1976), pp. 175–188. DOI: 10.1016/0021-9681(76)90044-8.

[19] Neuenschwander, B., Branson, M., and Spiegelhalter, D. J. "A note on the power prior". In: *Statistics in Medicine* 28.28 (2009), pp. 3562–3566. DOI: 10.1002/sim.3722.

[20] Banbeta, A. et al. "Modified power prior with multiple historical trials for binary endpoints". In: *Statistics in Medicine* 38.7 (2019), pp. 1147–1169. DOI: 10.1002/sim.8019.

[21] Gelman, A. "Prior distributions for variance parameters in hierarchical models (comment on article by Browne and Draper)". In: (2006). DOI: 10.1214/06-ba117a.

[22] Cunanan, K. M. et al. "Variance prior specification for a basket trial design using Bayesian hierarchical modeling". In: *Clinical Trials* 16.2 (2019), pp. 142–153. DOI: 10.1177/1740774518812779.

[23] Röver, C. and Friede, T. "Meta-analytic-predictive priors based on a single study". In: *arXiv preprint arXiv:2505.15502* (2025). DOI: 10.1017/rsm.2026.10081.

[24] Dalal, S. and Hall, W. "Approximating priors by mixtures of natural conjugate priors". In: *Journal of the Royal Statistical Society: Series B (Methodological)* 45.2 (1983), pp. 278–286. DOI: 10.1111/j.2517-6161.1983.tb01251.x.

[25] Diaconis, P. and Ylvisaker, D. "Quantifying Prior Opinion". In: *Bayesian Statistics 2: Proceedings of the Second Valencia International Meeting*. Ed. by Bernardo, J. M. et al. Amsterdam: North-Holland, 1985, pp. 133–156.

[26] Weru, V. et al. "Information borrowing in Bayesian clinical trials: choice of tuning parameters for the robust mixture prior". In: *Statistics in Biopharmaceutical Research* (Mar. 2026), pp. 1–21. ISSN: 1946-6315. DOI: 10.1080/19466315.2026.2646537. URL: http://dx.doi.org/10.1080/19466315.2026.2646537.

[27] Chen, N. and Lee, J. J. "Bayesian cluster hierarchical model for subgroup borrowing in the design and analysis of basket trials with binary endpoints". In: *Statistical Methods in Medical Research* 29.9 (2020), pp. 2717–2732. DOI: 10.1177/0962280220910186.

[28] Fujikawa, K. et al. "A Bayesian basket trial design that borrows information across strata based on the similarity between the posterior distributions of the response probability". In: *Biometrical Journal* 62.2 (2020), pp. 330–338. DOI: 10.1002/bimj.201800404.

[29] Held, L. and Sabanés Bové, D. *Applied Statistical Inference: Likelihood and Bayes*. Springer Berlin Heidelberg, 2014. ISBN: 9783642378874. DOI: 10.1007/978-3-642-37887-4. URL: http://dx.doi.org/10.1007/978-3-642-37887-4.

[30] Morita, S., Thall, P. F., and Müller, P. "Determining the effective sample size of a parametric prior". In: *Biometrics* 64.2 (2008), pp. 595–602. DOI: 10.1111/j.1541-0420.2007.00888.x.

[31] Hobbs, B. P., Carlin, B. P., and Sargent, D. J. "Adaptive adjustment of the randomization ratio using historical control data". In: *Clinical Trials* 10.3 (2013), pp. 430–440. DOI: 10.1177/1740774513483934.

[32] Neuenschwander, B. et al. "Predictively consistent prior effective sample sizes". In: *Biometrics* 76.2 (2020), pp. 578–587. DOI: 10.1111/biom.13252.

[33] Wiesenfarth, M. and Calderazzo, S. "Quantification of prior impact in terms of effective current sample size". In: *Biometrics* 76.1 (2020), pp. 326–336. DOI: 10.1111/biom.13124.

[34] Sung, L. et al. "Seven items were identified for inclusion when reporting a Bayesian analysis of a clinical study". In: *Journal of Clinical Epidemiology* 58.3 (2005), pp. 261–268. DOI: 10.1016/j.jclinepi.2004.08.010.

[35] Spiegelhalter, D. J. et al. "Bayesian methods in health technology assessment: a review". In: *Health Technology Assessment* 4 (2000). DOI: 10.3310/hta4380.

[36] Kruschke, J. K. "Bayesian analysis reporting guidelines". In: *Nature Human Behaviour* 5.10 (2021), pp. 1282–1291. DOI: 10.1038/s41562-021-01177-7.
